# Small bowel obstruction due to internal herniation through a right side diaphragmatic defect; an unusual case report

**DOI:** 10.1016/j.ijscr.2024.109831

**Published:** 2024-05-28

**Authors:** Fareed Ahmad Nazari

**Affiliations:** Department of Emergency Surgery & Trauma, Kabul University of Medical Sciences, Kabul, Afghanistan

**Keywords:** Diaphragmatic hernia, Bowel obstruction, Strangulation, Rare case

## Abstract

**Introduction:**

Right-side diaphragmatic hernia is a very rare cause of bowel obstruction and strangulation in adults, which is usually a congenital disorder.

**Case presentation:**

A- 55-year-old male presented to the emergency department of our hospital complaining of abdominal pain, nausea, vomiting, abdominal distension, fever, and constipation for 4 days. On physical examination, the patient was fibril, toxic, tachycardic, and hypotensive. The patient had a distended abdomen with exaggerated bowel sounds, abdominal tenderness, guarding, and rigidity mostly in the right upper quadrant. There were some degrees of tempanicity on percussion. The digital rectal examination was normal with no evidence of impacted stool.

**Discussion:**

Patients with a diaphragmatic hernia frequently present with manifestations of internal herniation, incarceration, obstruction, ischemia from strangulation, or perforation. The patient may present with respiratory symptoms such as dyspnea, absence of breath sounds in the thorax, or abdominal symptoms such as abdominal pain and bowel dilatation.

**Conclusion:**

Diaphragmatic hernia, which is a rare case, should be included in the differential diagnosis of small bowel obstruction to preclude complications.

## Highlights

• Right side diaphragmatic hernia is a very rare cause of bowel obstruction.
• It is usually a congenital disorder.
• Early diagnosis and treatment may preclude the complications.

## 1 Introduction

Right-side diaphragmatic hernia as a cause of bowel obstruction and strangulation of the affected bowel in adults is very rare [1]. Although bilateral diaphragmatic hernia is usually a congenital disorder, the unilateral spontaneous diaphragmatic hernia is usually caused by trauma, but its presence without any apparent history of trauma is even rarer and it is difficult to diagnose [2]. There are four types of congenital diaphragmatic hernia; hiatal hernia, para esophageal hernia, Bochdalek hernia, and Morgagni-Larrey hernia [3]. Diaphragmatic rupture is an infrequent consequence of trauma, occurring in about 1.13.9 % of severe blunt thoracoabdominal injuries in which abdominal contents herniate in less than half of the patients [4]. Acquired diaphragmatic hernias can be caused by all types of trauma (blunt, penetrating, or can be spontaneous in rare cases), but blunt trauma accounts for the majority of cases. Such hernias are mostly seen on the left rather than the right side as the liver provides a cushion effect on the right side, and the patient can present years after the initial trauma [4]. We present herein a rare case of right-side diaphragmatic hernia that manifested as bowel obstruction, small bowel ischemia, and subsequent strangulation, which required emergency laparotomy, bowel resection, anastomosis, and repair of the diaphragmatic defect. It is a surgical rare case report and has been reported in line with the SCARE criteria [5].

## 2 Case presentation

**Fig. 1:**
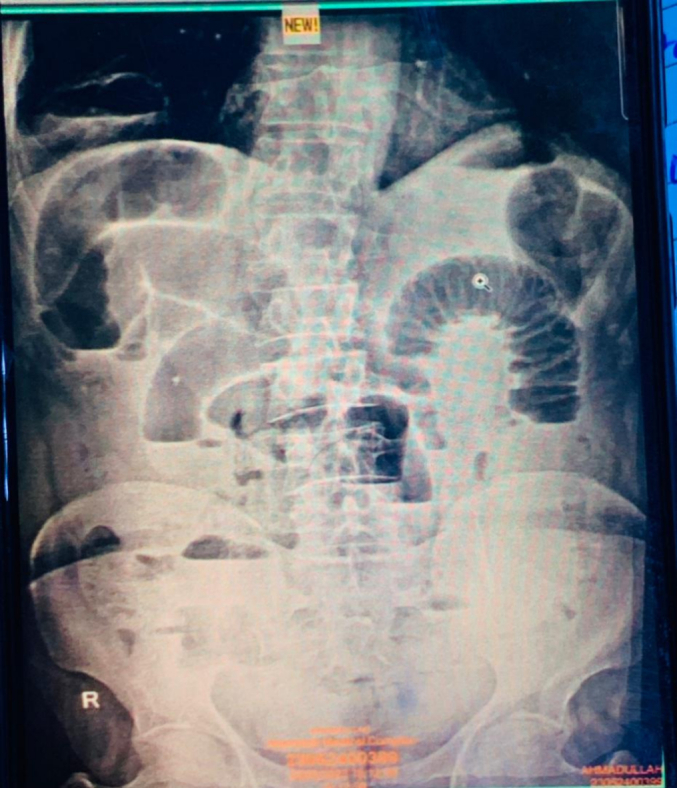
Radiograph of the abdomen and lower part of the chest in a standing position showing dilated bowel loops, and multiple air-fluid levels suggestive of small bowel obstruction. A loop of small bowel is also seen inside the chest above the right hemi-diaphragm.

**Fig. 2:**
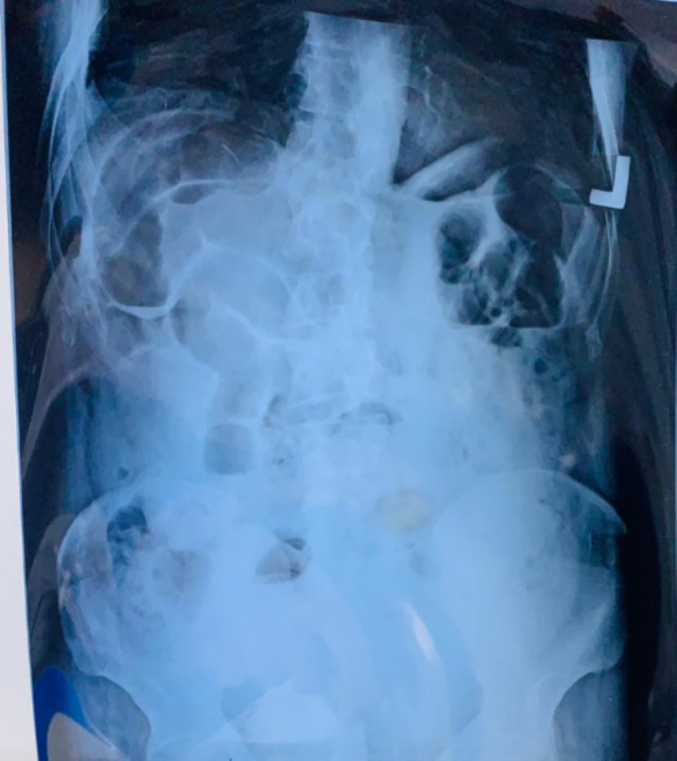
Radiograph of the abdomen and lower part of the chest in a standing position showing dilated bowel loop and air-fluid level suggestive of small bowel obstruction.

A 55-year-old male presented to the emergency department of the hospital with chief complaints of abdominal pain, nausea, vomiting, abdominal distension, fever, and constipation for 4 days. There no respiratory symptoms. The history of the patient was unremarkable, with no apparent thoracic or abdominal injury remembered by the patient. On examination, the patient was fibril, toxic, tachycardic, hypotensive, and suffering from abdominal pain. The abdomen was distended, bowel sounds were exaggerated, and abdominal tenderness, guarding, and rigidity were present, mostly in the right upper quadrant (RUQ). There were some degrees of tempanicity on percussion. Digital rectal examination (DRE) was normal with no evidence of impacted stool. Blood investigations revealed leukocytosis with a shift to the left. Hemoglobin, platelets, urea, creatinine, and liver function tests (LFTs) were within the normal limits. Ultrasound of the abdomen was unremarkable. Abdominal radiography in a standing position revealed dilated bowel loops and multiple air-fluid levels (Fig. 1, Fig. 2).

**Fig. 3:**
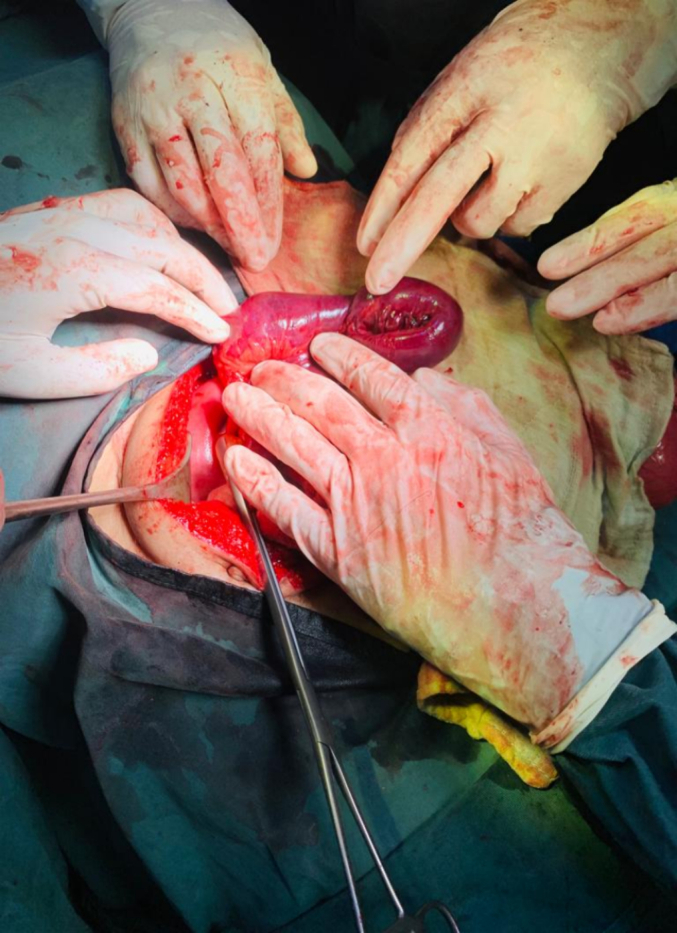
Intra-operative photograph showing a loop of jejunum entering the defect of the diaphragm; there is evidence of necrosis of a small part of the loop.

**Fig. 4:**
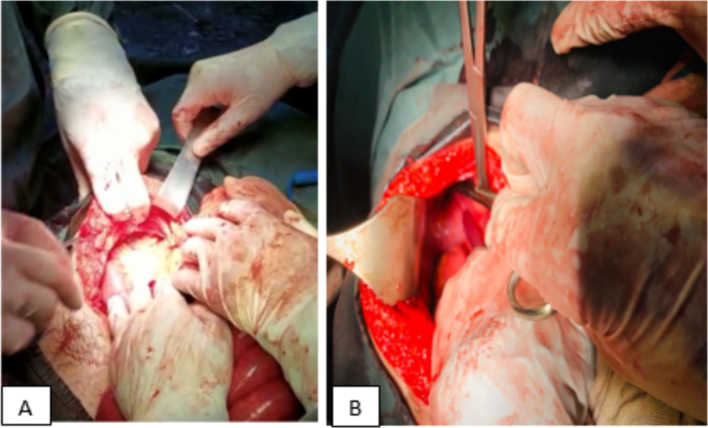
(A and B): Intraoperative photographs showing a loop of jejunum entering the defect of the diaphragm.

The patient was admitted with an initial diagnosis of bowel obstruction based on the abdominal radiographic findings. Following initial resuscitation, the patient was transferred to the operating room. After general anesthesia and tracheal intubation, the abdomen was opened by a midline incision. Intra-operatively, it was found that a loop of the jejunum pulled upward under the right hemidiaphragm, surrounded by the omentum, associated with proximal dilated bowls and collapsed colons. There was a defect in the border of the muscular part and tendonous part of the right hemidiaphragm measuring 2 × 2 cm, containing a loop of small bowel and mesentery (Fig. 3). The bowls were reduced back into the abdomen, however, there was evidence of strangulation (discoloration, absent distal mesenteric pulse, and absent peristalsis after stimulation in the affected loop) without perforation of the affected bowel (Fig. 4). The gangrenous bowel was resected and primary side to side anastomosis in two layers was performed. The diaphragmatic defect was closed by prolene. The draining tubes were placed in the Douglas pouch, subhepatic region along with tube thoracostomy. The abdomen was closed in layers. The bowel sounds were monitored postoperatively and the patient passed flatus on postoperative day 3. He was allowed orally on postoperative day 4 and was able to pass stools. The subhepatic, Douglas pouch drains, and thoracostomy tubes were removed on day 5. Subsequent chest radiography was unremarkable with no pneumothorax, hemothorax, or empyema formation. The patient was discharged home with full recovery.

## 3 Discussion

Diaphragmatic hernias has two types; congenital and acquired. Acquired diaphragmatic hernias (ADH) may be traumatic or iatrogenic in origin [6]. Traumatic ADH is usually due to a penetrating or blunt trauma and is undiagnosed in up to 33 % of cases during the immediate post-traumatic period [7]. In asymptomatic patients with thoracoabdominal stab wounds, the risk of an occult diaphragmatic injury is approximately 7 % and a mortality rate of 36 % following delayed diagnosis. Approximately 68.5 % of these injuries occur on the left side, 24.2 % on the right side, and 1.5 % occur on both sides [6]. The left-sided predominance is thought to be due to the relative weakness of the left hemidiaphragm and the protective effect of the liver on the right side [8]. As our patient had no history to support a traumatic diaphragmatic hernia, this was most likely congenital in etiology. The location of the diaphragmatic hernia was consistent with a Morgagni hernia.

Patients with diaphragmatic hernia frequently present with manifestations of visceral herniation, incarceration, obstruction, ischemia from strangulation, or perforation [9]. The patient may present with respiratory symptoms such as dyspnea, absence of breath sounds in the thorax, or abdominal symptoms such as abdominal pain and bowel dilatation [10]. Although respiratory symptoms in a patient presenting with bowel obstruction are usually secondary to aspiration, this case demonstrates the importance of considering a strangulated diaphragmatic hernia within the differential diagnosis [11].

Diagnosis of diaphragmatic hernia is performed using diagnostic modalities including plain radiography of the chest and abdomen, upper gastrointestinal contrast studies, diagnostic peritoneal lavage, fluoroscopy, ultrasound of the abdomen, computed tomography (CT-scan) of the abdomen, magnetic resonance imaging (MRI), laparotomy, and video-assisted thoracic surgery [12]. There is no single definite diagnostic tool for diagnosing diaphragmatic hernias. Surprisingly, only 25–50 % of initial chest films are diagnostic [13]. However, the diagnosis can be confirmed by CT-scan of abdomen [14].

The treatment of diaphragmatic hernia is surgery [2]. There is controversy as to whether a laparotomy or thoracotomy should be done; but in cases like this case where a patient presents with an acute abdomen and bowel obstruction without any respiratory manifestations, laparotomy is mandatory. Thoracotomy enables the division of the adhesions between thoracic and herniated abdominal viscera while in laparotomy bowel resection and anastomosis, if needed, are easy to be performed. Sometimes a combined approach may be necessary. The hernia contents are the small intestine mostly and may be a portion of the large intestine. The incarcerated loops of the bowel should be carefully dealt with. Diaphragmatic defects should be repaired by non-absorbable sutures or synthetic grafting [11]. More recently, successful laparoscopic and thoracoscopic repairs of the hernia have both been described. Some authors have also described hand-assisted thoracoscopic repair of hernia [4].

## 4 Conclusion

Diaphragmatic Hernia Presenting as Bowel Obstruction and Strangulation is an uncommon entity. However, it should be included in the differential diagnosis of small bowel obstruction with or without associated respiratory symptoms to preclude complications.

## Consent

Written informed consent was obtained from the patient for publication of this case report and accompanying images. A copy of the written consent is available for review by the Editor-in-Chief of this journal on request.

## Provenance and peer review

Not commissioned, externally peer-reviewed.

## Ethical approval

This report does not contain any personal information that could lead to the identification of the patient. Therefore, it is exempt from ethical approval.

## Funding

The authors declare that his work is not funded by any institution, organ, or government and he has no financial support.

## Guarantor

The corresponding author is the guarantor of submission.

## Research registration number

Not applicable.

## CRediT authorship contribution statement

Not applicable.

## Declaration of competing interest

The authors have no potential conflicts of interest to disclose.
